# *Astragalus* Polysaccharide Suppresses Doxorubicin-Induced Cardiotoxicity by Regulating the PI3k/Akt and p38MAPK Pathways

**DOI:** 10.1155/2014/674219

**Published:** 2014-10-16

**Authors:** Yuan Cao, Yang Ruan, Tao Shen, Xiuqing Huang, Meng Li, Weiwei Yu, Yuping Zhu, Yong Man, Shu Wang, Jian Li

**Affiliations:** ^1^The Fifth Clinical Hospital of Peking University, Health Science Center, Beijing 100730, China; ^2^Key Laboratory of Geriatrics, Beijing Hospital, Beijing Institute of Geriatrics, Ministry of Health of China, Beijing 100730, China; ^3^Capital Medical University Affiliated Beijing Anzhen Hospital, Beijing Institute of Heart, Lung and Blood Vessel Diseases, Beijing 100029, China

## Abstract

*Background*. Doxorubicin, a potent chemotherapeutic agent, is associated with acute and chronic cardiotoxicity, which is cumulatively dose-dependent. *Astragalus* polysaccharide (APS), the extract of *Astragalus membranaceus* with strong antitumor and antiglomerulonephritis activity, can effectively alleviate inflammation. However, whether APS could ameliorate chemotherapy-induced cardiotoxicity is not understood. Here, we investigated the protective effects of APS on doxorubicin-induced cardiotoxicity and elucidated the underlying mechanisms of the protective effects of APS. *Methods*. We analyzed myocardial injury in cancer patients who underwent doxorubicin chemotherapy and generated a doxorubicin-induced neonatal rat cardiomyocyte injury model and a mouse heart failure model. Echocardiography, reactive oxygen species (ROS) production, TUNEL, DNA laddering, and Western blotting were performed to observe cell survival, oxidative stress, and inflammatory signal pathways in cardiomyocytes. *Results*. Treatment of patients with the chemotherapeutic drug doxorubicin led to heart dysfunction. Doxorubicin reduced cardiomyocyte viability and induced C57BL/6J mouse heart failure with concurrent elevated ROS generation and apoptosis, which, however, was attenuated by APS treatment. In addition, there was profound inhibition of p38MAPK and activation of Akt after APS treatment. *Conclusions*. These results demonstrate that APS could suppress oxidative stress and apoptosis, ameliorating doxorubicin-mediated cardiotoxicity by regulating the PI3k/Akt and p38MAPK pathways.

## 1. Introduction

Anthracyclines such as doxorubicin, daunomycin, epirubicin, and idarubicin are anticancer chemotherapeutic drugs that are widely used in clinical cancer treatment [1]. Unfortunately, these types of drugs are associated with dose-dependent acute or chronic cardiotoxicity, which is characterized by hypotension, tachycardia, arrhythmia, transient depression of left ventricular function, and even refractory late-onset cardiomyopathy [2]. Because of such negative effects, their usage is limited despite their potent and effective functions in treating cancer. Doxorubicin has been postulated to induce cardiotoxicity because of its free radical-induced mitochondrial damage [3]. Given a series of studies reporting that reactive oxygen species (ROS) scavengers fail to effectively prevent cardiac toxicity, this hypothesis is not sufficient for explaining the cardiac toxicity caused by doxorubicin [4]. Many studies have demonstrated that doxorubicin induces DNA damage, inhibits DNA and protein synthesis, promotes myofiber degeneration, suppresses the transcription of specific genes, and results in cardiomyocyte apoptosis through a caspase-3-dependent mechanism [5].

The dry roots of* Astragalus membranaceus*, which is also known as* Huang Qi* in China and belongs to the Fabaceae family, have long been used as an important component of many herbal prescriptions in traditional Chinese medicine [6, 7].* Astragalus* species derivatives are crude drugs that have been widely used in traditional Chinese medicine for thousands of years as antiperspirant, antihypertensive, diuretic, and tonic treatments [8]. Studies from our group and others have indicated that* Astragalus* polysaccharide (APS), the extract from* Astragalus membranaceus*, exerts strong antitumor and antiglomerulonephritis activity [9] and effectively alleviates inflammation-induced artery endothelium cell injury and atherosclerosis [7]. Moreover, APS is used to ameliorate the side effects of doxorubicin treatment in cancer patients. However, whether APS could reverse chemotherapy-induced cardiotoxicity is not understood.

In stressed cardiomyocytes, such as those that occur during oxidative stress or ischemia reperfusion, the p38MAPK and PI3K/Akt pathways are altered. Cardiomyocyte loss during heart failure in the form of apoptotic cell death mainly contributes to deteriorating cardiac contractile function and left ventricular remodeling. Strong evidence has suggested that Akt activation could protect heart function by inhibiting apoptosis [10], and p38MAPK inhibition could attenuate cellular inflammatory reactions.

In this study, we investigated the protective effects of APS on doxorubicin-induced cardiotoxicity in cultured primary neonatal rat ventricular myocytes (NRVMs) and in C57BL/6J mice and elucidated the underlying mechanisms of the protective effects of APS. Our results demonstrate that APS could suppress oxidative stress and apoptosis and ameliorate doxorubicin-induced cardiotoxicity by regulating the PI3k/Akt and p38MAPK pathways. These findings provide insight into the mechanisms by which APS acts as a potential therapeutic prescription for lessening the cardiotoxicity caused by doxorubicin.

## 2. Materials and Methods

### 2.1. Clinical Data Retrospective Analysis

We performed a retrospective analysis of the clinical records of 206 patients with cancer including 73 patients with small cell lung cancer, 95 patients with breast cancer, 13 patients with Hodgkin lymphoma, and 25 patients with non-Hodgkin lymphoma (age: 49.9 ± 10.3 years; 45.78% men) who underwent doxorubicin chemotherapy (cumulative dose: 400–600 mg/m^2^) and had initial doxorubicin chemotherapy at Capital Medical University Affiliated Beijing Anzhen Hospital and Beijing Hospital between 2009 and 2013. Patient characteristics, including age, tumor type, tumor grade, chemotherapy received, and number of patients with cardiotoxicity, were recorded. Patients with comorbid conditions, such as diabetes, hypertension, and ischemic heart disease, were excluded; electrocardiographic, echocardiographic, and serial determinations of cardiac enzymes before and after doxorubicin chemotherapy were analyzed.

### 2.2. Animals

Eight-week-old male C57BL/6 mice were provided by Peking University Health Science Center. The mice (*n* = 60) were randomly divided into 3 groups of 20 mice each. Normal control (NC) mice orally received an equivalent volume of placebo (saline). Doxorubicin-treated mice (DOX) were injected with a single dose of doxorubicin dissolved in normal saline (20 mg/kg i.p.) and received an orally equivalent volume of saline. Doxorubicin plus APS treatment mice (DOX + APS) were pretreated with APS (1.5 g/kg) for 3 days by gavage and administered APS for 3 additional days after the injection of the same dose doxorubicin as the DOX group. The dosage of DOX and APS was modified according to previous studies [11, 12]. All of the mice in the 3 groups were euthanized 5 days after the initial injection of doxorubicin. All animal experiments conformed with the protocols approved by Beijing Hospital, the Ministry of Health Animal Use and Care Committee, and the Guide for Care and Use of Laboratory Animals (NIH Publication # 85-23, revised 1996).

### 2.3. Preparation of APS

APS was purchased from the ShiFeng Biological Co., Shanghai, China. The APS was dissolved in PBS to 10 mg/mL and then diluted with DMEM culture medium containing 10% FBS at different concentrations.

### 2.4. Reagent

Caspase 9, phosphorylated p38, phosphorylated Akt, and Bcl2 antibodies were purchased from Cell Signaling Technology (Danvers, MA, USA); caspase 3 antibody was purchased from Cell Signaling and Santa Cruz Biotechnology (Santa Cruz, CA, USA); secondary antibodies directed against rabbit or goat were purchased from Cell Signaling Technology. Unless otherwise indicated, all chemicals were purchased from Sigma (St. Louis, MO, USA).

### 2.5. Echocardiography

Mice were lightly anesthetized with 1–1.5% isoflurane in oxygen until the heart rate stabilized to 400 to 500 beats per minute. Echocardiography was performed using Vevo 770 and Vevo 2100 (VisualSonics) instruments. Fraction shortening (FS), ejection fraction (EF), left ventricular internal diameter (LVID) during systole, LVID during diastole, end-systolic volume, and end-diastolic volume were calculated with Vevo Analysis software (version 2.2.3) as previously described [13]. After echocardiography measurements, mice were euthanatized by cervical dislocation, and their hearts were collected for further analyses.

### 2.6. Histology, Immunofluorescence, and Immunohistochemistry

Histology and immunofluorescence assays were performed with hearts and sections as previously described [13]. Tissues were processed as cryosections and subsequently analyzed by H&E staining according to the manufacturer's protocol (Sigma-Aldrich). For the histological analysis, 8 *μ*m sections were incubated with primary antibodies overnight at 4°C. Afterward, the sections were washed with 0.25% Triton X-100 in PBS; the sections were incubated with either fluorescently labeled (Molecular Probes; Invitrogen) or biotinylated secondary (Vector) antibodies for 2 h. Finally, the sections were imaged by microscopy.

### 2.7. Terminal Deoxynucleotidyl Transferase-Mediated dUTP Nick End Labeling (TUNEL) and Hoechst 33342 Staining of Heart Cryosections and Cultured Cardiac Myocytes

Nuclear fragmentation was detected by TUNEL staining with an apoptosis detection kit (Roche) or by incubating fixed cells in 10 mM Hoechst 33342 as previously described [13]. Cells (500–700) in 10 randomly chosen fields from each dish were counted to determine the percentage of apoptotic nuclei. Each data point indicates results from 1600 to 2000 cells from 4 independent experiments [13].

### 2.8. Isolation and Culture of Rat Cardiac Myocytes

Neonatal rat ventricular myocytes (NRVMs) were isolated from 1–3-day-old Sprague Dawley rats via combined trypsin and collagenase type II digestion [13]. The cardiac myocytes were plated at a density of 6.6 × 10^4^ cells/cm^2^ in DMEM supplemented with 10% FBS in the presence of 0.1 mM 5-bromo-2-deoxyuridine.

### 2.9. Western Blotting Analysis

Cell lysates were analyzed by SDS-PAGE and electrotransferred to PVDF membranes. The membranes were blocked with 8% bovine serum albumin for 2 h and incubated with specific antibodies for 2 h. After 5 washes in TBST (TBS containing 0.1% Tween 20), the membranes were incubated with horseradish peroxidase-conjugated secondary antibodies in TBST for 1 h. Bands were detected by chemiluminescence detection reagents. Blot densitometry was performed, and bands were analyzed with ImageJ software.

### 2.10. Cell Viability Assay

Cell viability was examined by the MTT assay according to the instructions of the manufacturer. NRVMs (5000 cells/well) were plated in 24-well plates. NRVMs were pretreated with APS for 1 h and then treated with the indicated concentrations of DOX for 24 h. All assays were performed in triplicate. The cells were incubated with 0.5 mg/mL 3-[4,5-dimethylthiazol-2-yl]-2,5-diphenyltetrazolium bromide for 4 h, and the absorbance at 490 nm was measured as previously described [13]. The MTT kit was purchased from Roche Applied Science (Indianapolis, IN).

### 2.11. In Situ Detection of Reactive Oxygen Species (ROS)

To evaluate heart ROS production in situ, frozen, unfixed, whole heart cross-sections or living NRVMs were stained with 10 *μ*mol/L DHE (Sigma) for 30 min in a dark humidified chamber at 37°C. ROS generation was indicated by red fluorescence and visualized with fluorescence microscopy.

### 2.12. DNA Laddering

Cells were lysed in lysis buffer (10 mM Tris-Cl pH 8.0, 150 mM NaCl, 10 mM EDTA, 0.4% SDS, and 100 g/mL protease K) and incubated at 50°C for 5 h with gentle agitation. DNA was then extracted with phenol/CHCl_3_/isoamyl alcohol and CHCl_3_/isoamyl alcohol. DNA fragmentation was detected by loading 10 *μ*g of total DNA into a 2% agarose gel; the gel was run in Tris-acetate-EDTA buffer and visualized by ethidium bromide staining [13].

## 3. Statistical Analysis

All statistical calculations were performed using GraphPad Prism 5 software. The data are expressed as the mean ± SEM. Student's *t*-test was used to compare two conditions, and one-way ANOVA with Bonferroni correction was used for multiple comparisons. Probability values less than 0.05 were considered significant.

## 4. Results

### 4.1. Doxorubicin Treatment Leads to Increased Levels of Serum Myocardial Enzymes Accompanied by Heart Dysfunction in Cancer Patients

**Figure 1:**
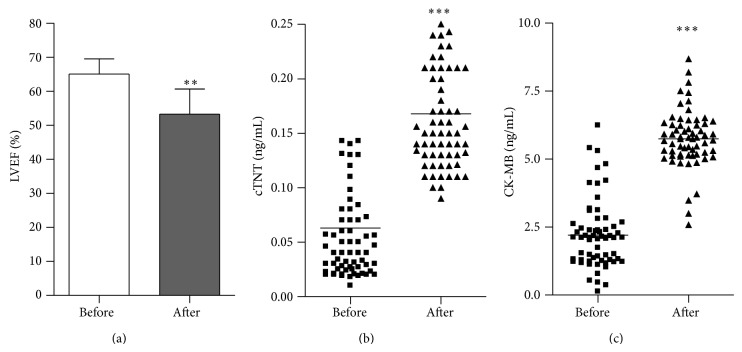
Increased levels of serum myocardial enzymes accompanied by heart dysfunction in cancer patients treated with doxorubicin. The average left ventricle ejection fraction (LVEF %) (a), median serum cTNT (b), and CK-MB (c) levels before and after chemotherapy (*n* = 63, ^**^ *P* < 0.01, ^***^ *P* < 0.001).

Clinical data from 206 cancer patients who had no comorbidities, such as diabetes, hypertension, and ischemic heart disease, and underwent initial doxorubicin-based chemotherapy (a cumulative dose of 450–600 mg/m^2^) were retrospectively analyzed. Fifteen patients who had no history of cardiovascular disease but an abnormal electrocardiograph (ECG) before chemotherapy were excluded from the database. A total of 63 patients (30.6% of total patients) who had a normal ECG before chemotherapy and an abnormal ECG after chemotherapy had a normal baseline LVEF 65.1 ± 4.5% and a final value less than 53.2 ± 7.4% after doxorubicin therapy (Figure 1(a)). Serial determinations of cardiac enzymes (cTNT, CK-MB) were performed. After chemotherapy, the median serum cTNT level increased from 0.04 ng/mL (75%—CI: 0.025–0.072 ng/mL) to 0.15 ng/mL (75%—CI: 0.13–0.21 ng/mL) for 63 patients (Figure 1(b)), and the median serum CK-MB level was elevated from 2.1 ng/mL (75%—CI: 1.29–2.49 ng/mL) to 5.73 ng/mL (75%—CI: 5.17–6.28 ng/mL) (Figure 1(c)). The average serum cTNT and CK-MB levels exceeded the threshold for normal values (0.1 and 5.0 ng/mL, resp.) [14].

### 4.2. Doxorubicin Induces Cardiomyocyte Injury by Promoting Oxidative Stress and Apoptosis

**Figure 2:**
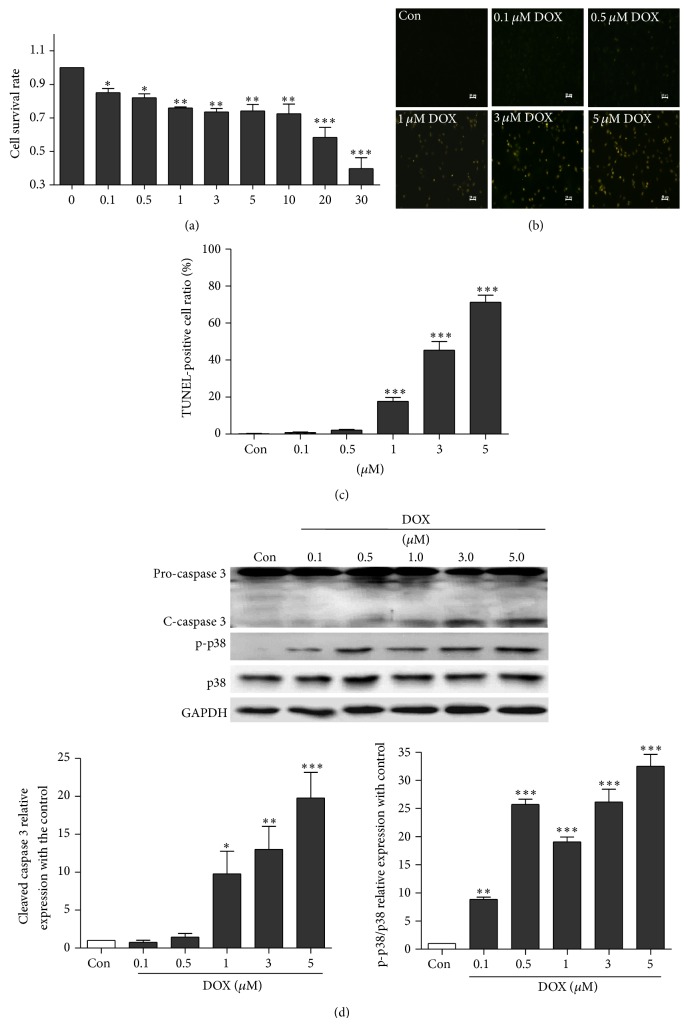
Doxorubicin induces cell injury by promoting apoptosis in cardiomyocytes. (a) The cell viability NRVMs cultured with different concentrations of doxorubicin (0, 0.1, 0.5, 1, 3, 5, 10, 20, and 30 *μ*M) for 24 h as demonstrated by MTT assay (*n* = 4). (b) Representative TUNEL staining of NRVMs cultured with different concentrations of doxorubicin (0, 0.1, 0.5, 1, 3, and 5 *μ*M) for 24 h. (c) Average TUNEL staining data (*n* = 4). (d) Dose response of activated (cleaved) caspase 3 and phosphorylated p38MAPK as assayed by Western blotting for NRVMs treated with 0.1–5.0 *μ*M doxorubicin for 24 h (*n* = 3) (^*^ *P* < 0.05, ^**^ *P* < 0.01, and ^***^ *P* < 0.001 versus the control group).

**Figure 3:**
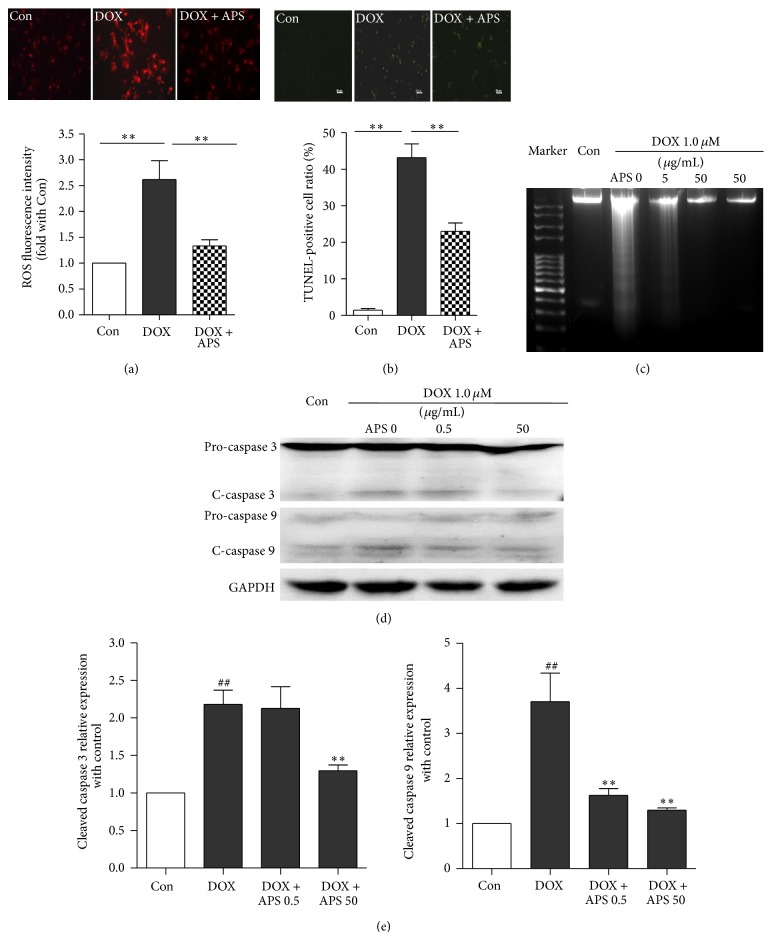
APS reverses the doxorubicin-induced oxidative stress and apoptosis of cardiomyocytes. (a) DHE staining of control NRVMs, doxorubicin-treated (1 *μ*M) NRVMs, and NRVMs pretreated with APS (50 *μ*g/mL) followed by doxorubicin treatment (*n* = 4, ^**^ *P* < 0.01). (b) Cardiomyocyte apoptosis as detected by TUNEL in control and doxorubicin-treated (1 *μ*M) NRVMs and NRVMs pretreated with APS (50 *μ*g/mL) followed by doxorubicin treatment (*n* = 4). (c) Cardiomyocyte apoptosis as detected by DNA laddering for control and doxorubicin-treated (1 *μ*M) NRVMs and NRVMs pretreated with APS (50 *μ*g/mL) followed by doxorubicin treatment (*n* = 4). (d) APS suppressed doxorubicin-induced caspase 3 and caspase 9 activation in a concentration-dependent manner in NRVMs as assayed by Western blotting (*n* = 3) (^##^ *P* < 0.01 versus control group, ^*^ *P* < 0.05 versus the doxorubicin-treated group, and ^**^ *P* < 0.01 versus the doxorubicin-treated group).

Cell viability assays were performed using doxorubicin-treated NRVMs. As shown in Figure 2(a), doxorubicin treatment reduced cell viability in a dose-dependent manner compared with the control; 0.1 *μ*M doxorubicin treatment led to 20% decreased cell viability. Similarly, TUNEL assay (Figure 2(b)) verified that doxorubicin triggered apoptosis in a dose-dependent manner. Moreover, to explore the underlying mechanisms of cardiotoxicity induced by doxorubicin, cell survival and apoptosis-related signaling proteins such as caspase 3 and p38MAPK were detected. The levels of active (cleaved) caspase 3 and phosphorylated p38MAPK were significantly increased in a concentration-dependent manner in cardiomyocytes treated for 24 h with doxorubicin (Figure 2(c)). Moreover, doxorubicin led to elevated ROS generation in the hearts of C57BL/6J mice and cultured NRVMs (Figure 3(a)). These results indicate that doxorubicin could induce cardiotoxicity via oxidative stress and apoptosis.

### 4.3. APS Reverses Doxorubicin-Induced Oxidative Stress and Apoptosis in Cultured Primary Neonatal Rat Ventricular Myocytes

As shown in Figure 3(a), NRVMs pretreated with 50 *μ*g/mL APS attenuated doxorubicin-induced ROS generation. APS pretreatment at different concentrations also reduced doxorubicin-mediated NRVM apoptosis as demonstrated by TUNEL staining and DNA laddering (Figures 3(b) and 3(c)). Moreover, Western blot analysis indicated that, after APS pretreatment, the level of cleaved caspase 3 caused by doxorubicin was decreased. Similarly, activation of caspase 9, a cysteine protease involved in the mitochondrial apoptosis pathway, was reduced in NRVMs pretreated with APS (Figures 3(d) and 3(e)). Taken together, these results demonstrate that APS could reverse doxorubicin-induced oxidative stress and apoptosis in cultured NRVMs.

### 4.4. APS Ameliorates Doxorubicin-Caused Cardiotoxicity and Apoptosis In Vivo

**Figure 4:**
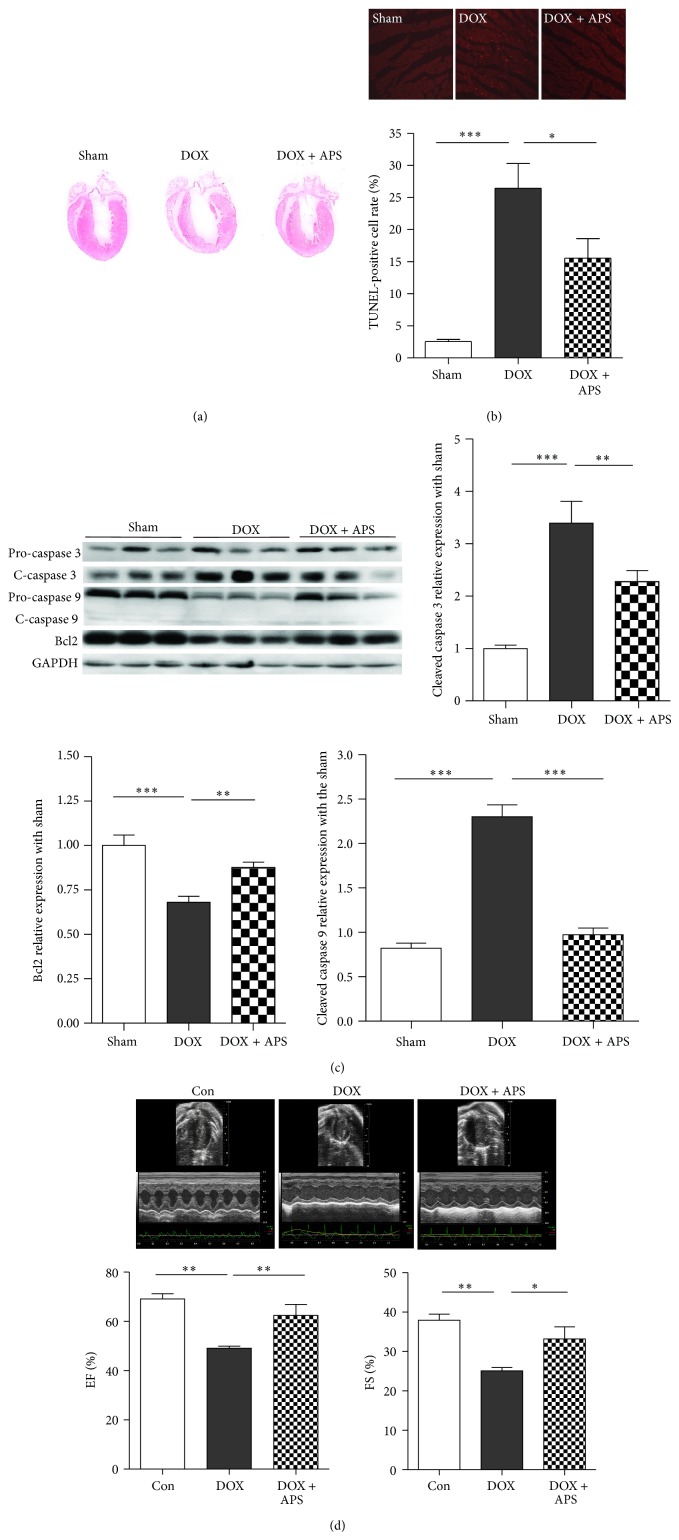
APS ameliorates doxorubicin-induced cardiotoxicity and apoptosis in vivo. (a) H&E staining of normal mice (Con) and mice with doxorubicin-induced heart failure (HF) (*n* = 5). (b) Representative TUNEL staining of apoptotic cells in normal and doxorubicin-induced heart injury samples. Red staining indicates TUNEL-positive cells (*n* = 5, ^*^ *P* < 0.05, ^**^ *P* < 0.01). (c) Western blotting and average data for caspase 3, caspase 9, and Bcl2 in sham, doxorubicin-induced heart injury mice (DOX), and mice with APS pretreatment followed by doxorubicin treatment (APS + DOX) (*n* = 15, ^**^ *P* < 0.01, ^***^ *P* < 0.001). (d) Mouse heart function 5 days after doxorubicin injection as shown by fractional shortening % (FS %) and ejection fraction % (EF %) (*n* = 8, ^*^ *P* < 0.05, ^**^ *P* < 0.01).

To determine whether APS could preserve heart function in vivo, we next generated a heart failure model using doxorubicin-treated C57BL/6 mice. As shown by H&E staining, doxorubicin-induced heart failure was associated with decreased thickening of the left ventricular wall and ventricular dilation (Figure 4(a)). The number of apoptotic cells was dramatically higher in hearts of doxorubicin-treated C57BL/6 mice (26.44 ± 7.72%) compared with hearts from control mice (2.55 ± 0.65%) as demonstrated by TUNEL staining. Importantly, pretreatment with APS attenuated doxorubicin-induced cardiomyocyte apoptosis (15.54 ± 6.06%) (Figure 4(b)). Moreover, Western blot analysis revealed that APS suppressed doxorubicin-induced caspase 3 and caspase 9 activation. In addition, Bcl2 protein expression was dramatically upregulated in APS pretreated mice (Figure 4(c)).

To further explore the clinical relevance of the protective effects of APS treatment on doxorubicin-induced heart failure, we measured heart function by Echo analysis. Compared with control mice, doxorubicin-treated mice exhibited decreased heart function as measured by the ejection fraction (EF) % and fraction shortening index (FS) % upon echocardiogram. However, pretreatment with APS significantly attenuated the reduction in heart function (Figure 4(d)). These data suggest that APS could protect heart function by suppressing doxorubicin-caused cardiotoxicity and apoptosis.

### 4.5. APS Attenuates Doxorubicin-Induced Heart Injury Regulating the p38MAPK and Akt Pathways

**Figure 5:**
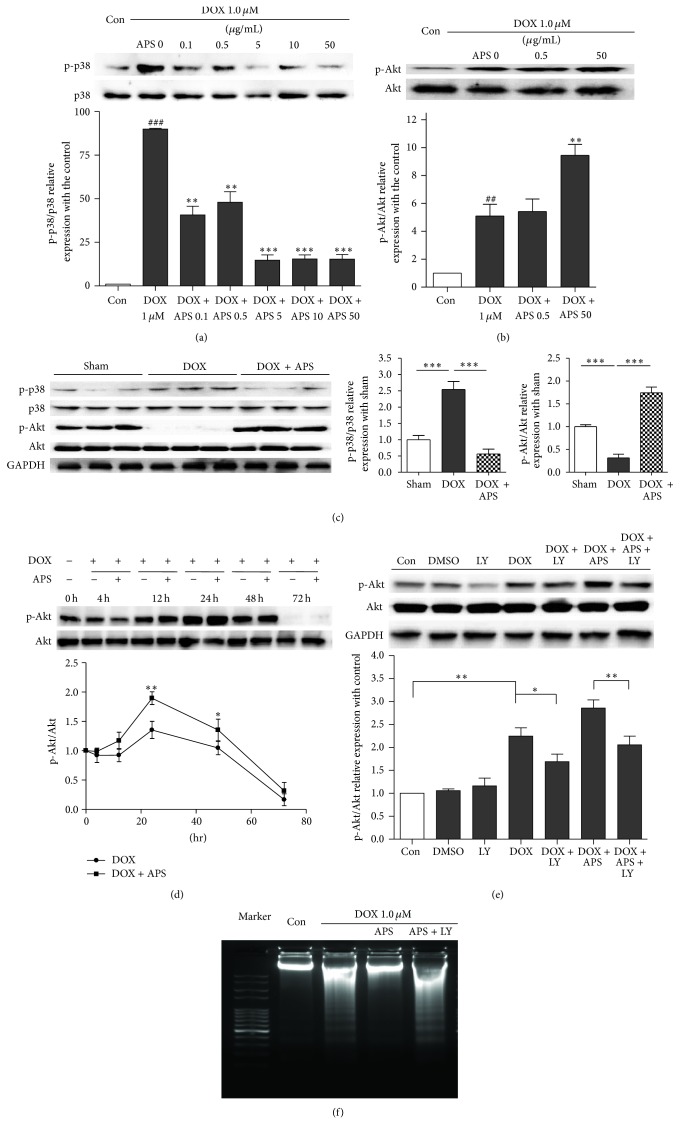
APS attenuates doxorubicin-induced heart injury by regulating p38MAPK and Akt phosphorylation. (a) APS decreases p38MAPK phosphorylation in a concentration-dependent manner in NRVMs as assayed by Western blotting (*n* = 3, ^###^ *P* < 0.001 versus control group, ^**^ *P* < 0.01 versus the doxorubicin-treated group, and ^***^ *P* < 0.001 versus the doxorubicin-treated group). (b) APS increases Akt phosphorylation in NRVMs (*n* = 3, ^##^ *P* < 0.01 versus control group, ^**^ *P* < 0.01 versus the doxorubicin-treated group). (c) Western blotting and average data for p38MAPK and Akt phosphorylation in sham and doxorubicin-induced heart injury mice and mice pretreated with APS (*n* = 8-9, ^***^ *P* < 0.001). (d) Time course of doxorubicin-induced Akt phosphorylation in NRVMs (*n* = 3, ^*^ *P* < 0.05 versus 0 hr sample, ^**^ *P* < 0.01 versus 0 hr sample). (e) The APS protective effect was reversed by the PI3K inhibitor LY294002. NRVMs were pretreated for 1 h with 2 *μ*M LY294002 prior to APS (50 *μ*g/mL) and DOX (1 *μ*M) treatment. Akt phosphorylation and cleaved caspase 3 were analyzed by Western blotting (*n* = 3, ^*^ *P* < 0.05, ^**^ *P* < 0.01). (f) Cardiomyocyte apoptosis as detected by DNA laddering in control and DOX-treated NRVMs, NRVMs pretreated with APS (50 *μ*g/mL) followed by DOX treatment, and NRVMs pretreated with APS (50 *μ*g/mL) and LY294002 (2 *μ*M) followed by DOX treatment (*n* = 4).

To further elucidate the underlying mechanisms responsible for the protective effects of APS on doxorubicin-induced heart injury, we analyzed changes in several important cell survival and death signaling pathways including the PI3K/Akt, ERK, p38MAPK, and JNK pathways in response to doxorubicin treatment and APS pretreatment. We found that pretreatment with APS significantly attenuates doxorubicin-induced p38MAPK phosphorylation in a concentration-dependent manner in NRVMs (Figure 5(a)). However, the ERK and JNK signaling pathways were not responsive to doxorubicin treatment and APS pretreatment (data not shown). Moreover, doxorubicin treatment increased the Akt phosphorylation in NRVMs, which was further increased by APS pretreatment (Figure 5(b)). After pretreatment with APS, in vivo Akt phosphorylation was dramatically enhanced, which corresponded with the results obtained in NRVMs. However, the Akt phosphorylation found with doxorubicin-induced mouse heart failure significantly decreased (Figure 5(c)).

We also analyzed the time course of doxorubicin-induced Akt phosphorylation in NRVMs. As shown in Figure 5(d), doxorubicin dramatically increased Akt phosphorylation beginning with the 12 h treatment, and this was reduced at 48 h and further lowered at 72 h. These data suggest that doxorubicin treatment for a short period could increase Akt phosphorylation as a feedback mechanism in NRVMs. C57BL/6 mice were treated with doxorubicin for 5 days. Thus, the heart Akt phosphorylation was significantly decreased.

In addition, we used LY294002 (2 *μ*M) [15], a PI3K inhibitor, to inhibit Akt phosphorylation. As expected, LY294002 significantly suppressed the elevated Akt phosphorylation caused by APS pretreatment (Figure 5(e)). Accordingly, DNA laddering showed that the APS-mediated protection of doxorubicin-treated NRVMs could be abrogated by LY294002 (Figure 5(f)). In summary, in vitro and in vivo results indicate that APS exerts its cardioprotective effects against doxorubicin-induced cardiomyocyte injury by regulating the p38MAPK and Akt pathways.

## 5. Discussion

### 5.1. Doxorubicin Triggers Cardiotoxicity In Vitro and In Vivo

The anthracycline drug doxorubicin remains one of the most potent active antineoplastic agents used for the treatment of solid tumors and hematologic malignancies [16]. However, acute and chronic cardiotoxicity limit its clinical use. It has been suggested that doxorubicin could cause severe cardiomyopathy and heart failure.

In this study, we collected the clinical information of 206 cancer patients who had no history of cardiovascular disease and performed a retrospective analysis. Approximately 30.6% of patients who had a normal ECG before chemotherapy and an abnormal ECG after chemotherapy had a decreased LVEF with a cumulative dose of 450–600 mg/m^2^. Our results are similar to the retrospective analysis of three trails performed by Swain et al., who reported that the estimated percentage of patients receiving doxorubicin-based treatment at a cumulative dose of 550 mg/m^2^ who had doxorubicin-induced heart injury is 26% [17].

In vitro and in vivo experiments have been previously performed using cardiomyocyte cell lines such as H9c2, neonatal rat ventricular myocytes, mice, and rats to investigate doxorubicin-induced cardiomyocyte injury. According to previous research, doxorubicin could induce cardiomyocyte loss and heart dysfunction in a dose-dependent manner [18, 19], which was confirmed by our study. Thus, considering potent effects for the treatment of solid tumors and hematologic malignancies [20–22], we should focus considerable attention on lessening DOX-induced cardiotoxicity, reducing the doxorubicin limit for clinical usage.

### 5.2. APS Protects Cardiomyocytes from Doxorubicin-Induced Oxidative Stress and Apoptosis

APS has long been known to act as an effective traditional medicine for enhancing immunity, inhibiting tumor growth, and alleviating inflammation-induced artery endothelium cell injury and atherosclerosis [9, 23]. Among these functions, APS has been most widely studied, mainly for its immunopotentiating properties such as stimulating immunocyte proliferation and cytokine production to defend against cancer [9]. Additionally, previous studies have demonstrated that APS may be used as a potent protective medicine for addressing heart diseases such as myocardial hypertrophy and heart failure [7]. Many Chinese patent medicines derived from APS are prescribed to patients with cardiac disease. Clinical studies have also indicated that APS could counteract the side effects of chemotherapeutic drugs, such as remarkably mitigating the degree of myelosuppression in cancer patients [11]. However, few groups have investigated whether APS could provide benefit for hearts damaged by doxorubicin treatment. In this study, we verified that APS markedly reduces doxorubicin-induced cardiomyocyte injury via the suppression of oxidative stress and apoptosis.

### 5.3. APS Suppresses Cardiomyocyte Apoptosis via Activation of the PI3K/Akt Signaling Pathway

Apoptosis is important for the development of most organs and adult tissue homeostasis and remodeling. However, inexorable loss of terminally differentiated cardiomyocytes followed by their replacement with fibrotic tissue is a hallmark of the transition from cardiac hypertrophy to functionally decompensated heart failure [24], thus playing a pivotal role in the development of various heart diseases.

Previous studies have shown that the PI3K/Akt signaling pathway provides an essential cell survival signal in cardiomyocytes [25]. In this study, we found that APS could activate the PI3K/Akt signaling pathway and reduce doxorubicin-induced cardiomyocyte apoptosis. Doxorubicin induced an increase in Akt phosphorylation, which is similar to findings by Swain et al. [17, 26], and considered a compensatory protective upregulation. However, our in vitro experiments showed that, after APS treatment, phosphorylated Akt was dramatically decreased. To explain the different phenomenon, we analyzed the time course of doxorubicin-induced Akt phosphorylation in NRVMs. Western blotting showed that phosphorylated Akt was elevated after doxorubicin treatment within a short period, which was reduced after 48 h. These results strongly support the explanation that doxorubicin treatment could induce a compensatory Akt phosphorylation increase within a short period [17, 27]. After APS pretreatment, phosphorylated Akt dramatically increased as compared with the DOX group, whereas proapoptotic proteins, such as activated caspase 3 and caspase 9, were decreased in vitro and in vivo, indicating that APS could effectively suppress apoptosis by activating the Akt pathway.

### 5.4. APS Impairs Doxorubicin-Induced ROS Generation and p38MAPK Activation in Cardiomyocytes

Myocardial oxidative stress has been shown to lead to ventricular dilatation in humans and animal heart failure models [28]. Doxorubicin has been suggested to induce the activation of p38MAPK, leading to inflammatory reactions and cell injury [29]. Consequently, reducing oxidative stress by lowering ROS production is crucial for the management of doxorubicin-induced cardiotoxicity. In this study, we found that doxorubicin led to increased ROS generation and p38MAPK activation, which was largely decreased by treatment with APS. Given that oxidative stress could activate p38MAPK, APS could effectively impair p38MAPK phosphorylation and attenuate cell inflammation, which suppresses cardiomyocyte apoptosis and injury in vivo and in vitro.

### 5.5. The Clinical Relevance of Heart Failure and APS Treatment

In this study, we demonstrated that pretreatment with APS suppresses the doxorubicin-induced apoptosis of NRVMs in vivo and in vitro. APS treatment efficaciously protects the heart from the side effects induced by doxorubicin treatment. Therefore, our findings may implicate novel drug targets and therapeutic strategies for doxorubicin-induced heart diseases, including myocardial infarction, dilated cardiomyopathy, and congestive heart failure.

However, before these findings can be applied to clinical medicine, several important issues must be addressed. First, although our study clearly indicates that APS protects cardiomyocytes from doxorubicin-induced apoptosis in cell and mouse models, it requires further investigation to determine its clinical effects and application. Second, given that APS has various pharmacological effects, our study, which focused on antiapoptotic and oxidative stress inhibition, is not thorough. Further studies are required to define the potential protective roles of APS in doxorubicin-induced cardiotoxicity.

## 6. Conclusions

In summary, we found that APS could protect the heart from doxorubicin-induced oxidative stress and apoptosis by inducing Akt phosphorylation and inhibiting p38MAPK phosphorylation. These findings provide insight into the mechanisms by which APS acts as a potential therapeutic prescription for lessening cardiotoxicity caused by doxorubicin.

## Significance

Our data demonstrate that APS markedly reduces doxorubicin-induced cardiomyocyte injury. APS pretreatment is sufficient to protect cardiomyocytes from doxorubicin-induced oxidative stress and apoptosis via its antioxidative stress and anti-inflammation effects.
